# Correlated Spectroscopy of Electric Noise with Color Center Clusters

**DOI:** 10.1021/acs.nanolett.4c00222

**Published:** 2024-05-20

**Authors:** Tom Delord, Richard Monge, Carlos A. Meriles

**Affiliations:** † Department of Physics, CUNY-City College of New York, New York, New York 10031, United States; ‡ CUNY-Graduate Center, New York, New York 10016, United States

**Keywords:** spectral diffusion, noise spectroscopy, charge traps, nitrogen-vacancy centers, diamond

## Abstract

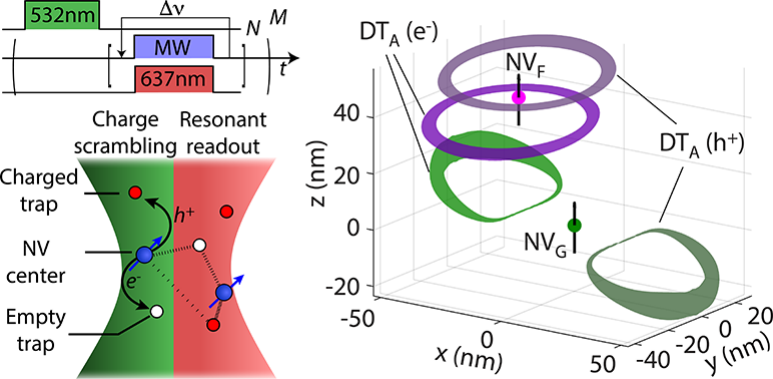

Experimental noise often contains information about the interactions of a system with its environment, but establishing a relation between the measured time fluctuations and the underlying physical observables is rarely apparent. Here, we leverage a multidimensional and multisensor analysis of spectral diffusion to investigate the dynamics of trapped carriers near subdiffraction clusters of nitrogen-vacancy (NV) centers in diamond. We establish statistical correlations in the spectral fluctuations we measure as we recursively probe the cluster optical resonances, which we then exploit to reveal proximal traps. Further, we deterministically induce Stark shifts in the cluster spectrum, ultimately allowing us to pinpoint the relative three-dimensional positions of interacting NVs as well as the location and charge sign of surrounding traps. Our results can be generalized to other color centers and provide opportunities for the characterization of photocarrier dynamics in semiconductors and the manipulation of nanoscale spin-qubit clusters connected via electric fields.

Often seen as detrimental, random fluctuations in the response of a probed system can nonetheless shed light on otherwise hidden physical processes. A paradigm example is Johnson noise, the intrinsic voltage fluctuations in a resistor, whose root-mean-square amplitude can be tied to the system temperature.^1^ Another illustration can be found in the statistical fluctuations of a paramagnetic ensemble,^2^ where random spin coherences forming and decaying with characteristic energies and time scales can be leveraged to obtain the system’s magnetic resonance spectrum—even in multidimensional form—without external drives.^3−5^

While fundamental fluctuations are detectable in macroscopic systems,^3,4^ experiments at the nanoscale are often better suited for noise spectroscopy because the fractional change on the observed random signal increases as the system size decreases.^6−8^ In particular, simultaneous correlations in space and time—recently proposed as a strategy to enhance the information content of quantum sensing experiments^9^—are intrinsically easier to capture in nm-sized systems.

Here we monitor the optical transitions of individual NVs within clusters sharing the same diffraction-limited volume as we randomly alter the occupation of proximal charge traps. Comparing the spectra from multiple measurements—impacted by reconfiguring electric environments—we extract single- and multi-NV spectral correlations, that we then use to map out the NV relative positions in three dimensions and co-locate proximal charge traps in the crystal host. Capitalizing on the dual role of NVs —alternatively serving as an electric field probe or a carrier trap—we further illustrate how controlled ionization of an individual center from a pair in the cluster allows us to deterministically change the optical resonances of the other. Related work has been reported recently, both for NVs in diamond^10^ and organic color centers in carbon nanotubes.^11^

## Fundamentals and Experimental Design

**Figure 1:**
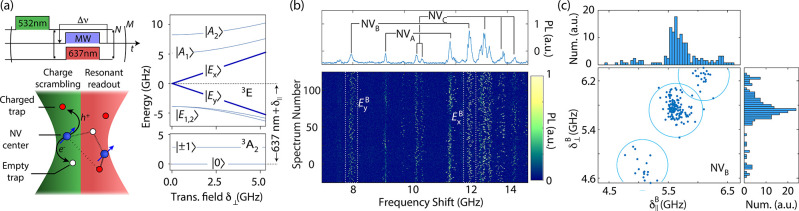
**Optical spectroscopy of diffraction-limited NV clusters.** (a) (Left) We use resonant confocal microscopy to individually address NVs from a small set sharing the same diffraction-limited volume. A 532 nm laser initializes the NV charge state and scrambles the charge environment before we reconstruct a full spectrum under simultaneous MW (2.88 GHz) and tunable 637 nm light. (Right) Under cryogenic conditions, the NV^–^ excited state manifold ^3^E splits into two triplets yielding a collection of optical resonances around 637 nm whose values depend on the electric and strain fields at each NV site. (b) Recursive photoluminescence excitation (PLE) spectroscopy of a cluster featuring three different NVs. The bottom plot shows successive spectra obtained from the sequence in (a). The upper 1D plot is the integrated sum of all individual traces; spectral-diffusion-induced broadening of all resonances is apparent. (c) Correlated longitudinal and transverse fields on NV_B_ as extracted from the highlighted resonances in the spectra in (b). Data clustering (indicated by circles) is apparent in the 2D plot but not so much in the 1D projected histograms (upper and right inserts). In (b), the green (red) laser power during charge initialization (frequency sweep) is 1 μW (3 nW) and the reference frequency is 470.470 THz. Unless otherwise noted, all experiments are carried out at 9 K in the absence of any externally applied magnetic field.

We used narrow-band, tunable laser excitation (637 nm) to monitor small sets of negatively charged NVs via confocal microscopy. Formed by a substitutional nitrogen adjacent to a vacancy,^12^ these spin-active color centers are attracting broad attention as a platform for quantum information processing^13^ and nanoscale sensing.^14^ At low temperatures, the zero-phonon line features a fine structure, best captured through the energy diagram in Figure 1a: Importantly, the ^3^E excited state manifold features a set of environment-sensitive optical resonances whose frequencies depend on the transverse and longitudinal components of the combined electric and strain fields^12^ (respectively, δ_⊥_ and δ_∥_). Undesirable to most applications,^13^ the large NV susceptibility is instead valuable herein, as it heralds reconfigurations in the occupation of proximal charge traps (Figure 1a). Though detection of single charges with the ground state NV spin is possible,^15−17^ resonant optical excitation dramatically improves its range. For example, a 10 MHz shift—grossly one line width of the optical and magnetic resonances—responds to the capture of a single electron 500 nm^18^ and 2 nm away, respectively.

We introduce our working strategy in Figure 1b where we plot a series of photoluminescence excitation (PLE) spectra from a first NV cluster. Combined use of selective NV^–^ ionization and microwave excitation allows us to identify three NVs within the same diffraction-limited volume (hereafter denoted A, B, and C, see also Supporting Information (SI), Sections 1–4). Spectral “diffusion” of the optical resonances is apparent as we intercalate pulses of green light (532 nm) between successive laser sweeps. The power and duration of these pulses—adjusted to cycle the NV charge state between negative and neutral—also lead to changes in the occupation of coexisting traps, hence resulting in a varying electric environment.

While fluctuations in trap occupation far from an NV induce a near-continuous diffusion of the optical spectrum, proximal charge state changes must arguably lead to discernible spectral jumps. These, in turn, can be leveraged to measure discrete changes in δ_⊥_ and δ_∥_ (respectively associated with shifts between optical resonances and of the entire multiplet,^18^Figure 1a), ultimately providing information not apparent in a time-averaged spectrum (upper insert in Figure 1b). We first demonstrate this notion in Figure 1c where we build on the spectra measured for NV_B_ to derive a two-dimensional (2D) field histogram correlating the values derived for δ_⊥_ and δ_∥_. We find a nonuniform distribution, which hints at discrete jumps along with a quasi-Gaussian electric noise background. Crucially, data bunching is far less obvious if one only considers each field projection separately (upper and right inserts in Figure 1c), which underscores the need for a correlated, multivariate analysis.

## Mapping out Proximal Charge Traps

**Figure 2:**
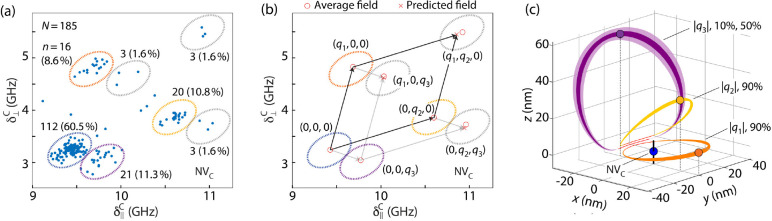
**Mapping out proximal charge traps.** (a) Longitudinal and transverse fields for NV_C_ as extracted from the spectroscopy set in Figure 1b; *N* indicates the total number of observations, and *n* is the number of data points enclosed in each ellipse. (b) We model the electric field on NV_C_ as the combined effect from point charges in three proximal traps and a fluctuating field environment of more distant carriers. The probability of a given charge configuration can be extracted from the fractional weight of each data cluster in the plot, where not more than one trap is populated. This information is sufficient to predict the fields and occurrence probability of those configurations in which more than one trap is occupied (see crosses within gray ellipses). (c) Probability isosurfaces for the point charge positions yielding the average fields shown in (b) for the case of electron-populated traps; percent values denote confidence intervals. Red lines indicate sections of otherwise possible solutions, here discarded based on observations with two simultaneously populated traps.

To better illustrate the advantages and limitations intrinsic to this class of noise spectroscopy, we resort to NV_C_ where the 2D field histogram of δ_⊥_ and δ_∥_ reveals a complex, though richer structure (Figure 2a). To interpret our observations, we model the electric field environment as the stochastic sum of contributions from carriers at three sites (Figure 2b,c). Assuming each trap intermittently hosts one fundamental charge, we converge to a set of four “primary” configurations corresponding to states where all traps are empty or where a carrier occupies one of the three possible sites.

One can combine these alternative scenarios to predict the transverse and longitudinal fields expected in cases in which more than one trap is occupied. We demonstrate this idea in Figure 2b where we add the fields produced by carriers in traps 1 and 2 (respectively, (*q*_1_, 0, 0) and (0, *q*_2_, 0)) to obtain the data subset in the upper right corner of the plot, (*q*_1_, *q*_2_, 0); it is easy to see the same applies to the two alternative cases (sites 1 and 3, or 2 and 3, faint vectors in Figure 2b). Since transverse components—only determined in magnitude—follow correctly from a vector sum, we conclude that all traps approximately lie on the same plane; further, because each trap hosts on average one carrier (13 ± 2)% of the time (Figure 2a), we expect to find two simultaneously full traps with a probability of only (2 ± 1)%, in agreement with our observations ((1.6 ± 1)%, gray ellipses in Figure 2a,b).

We can now utilize the known electric field susceptibility of the NV excited orbitals to gain information on the source charge locations; the caveat, however, is that changes in the transverse and longitudinal fields as measured from a single NV are insufficient to fully determine the 3D position and charge of a trap (SI, Sections 5–7). We plot in Figure 2c the calculated isosurfaces of position probability distributions as seen by NV_C_ (Figure 2a,b) assuming trapped electrons. Interestingly, we can exclude sections of the solution set that are incompatible with observations where more than one trap is populated (red segments in each loop in Figure 2c). We show below how this “co-measurement” can be adapted to cases where more than one NV picks up the field from a single trap.

## Multi-NV Correlated Spectroscopy

**Figure 3:**
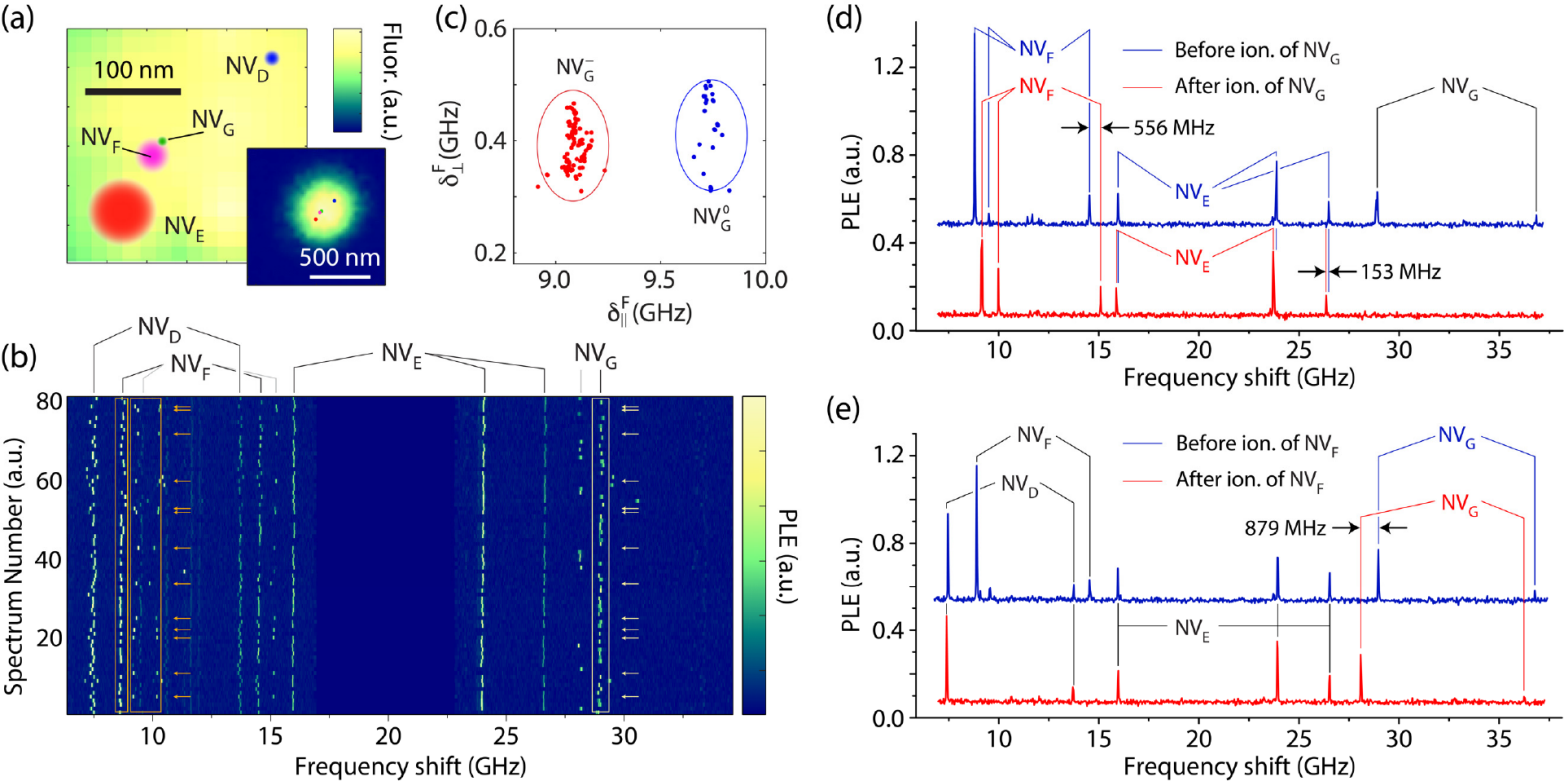
**Coulomb-field control of NV optical resonances.** (a) Confocal image (532 nm excitation) of a second cluster comprising four different NVs. Superimposed circles indicate the in-plane positions, as determined from confocal imaging using laser light resonant with the *E*_*y*_ transition of each NV in the set; the radius in each circle indicates the uncertainty. The lower right insert is a zoomed-out image of the same cluster. (b) Recursive optical spectroscopy of the NV cluster in (a). Individual inspection of the spectra in the series reveals a correlation between NV_G_ ionization (heralded by a missing optical resonance, yellow arrows) and the shift to longer wavelengths in the optical resonances of NV_F_ (orange arrows). All conditions are as in Figure 1b. (c) Correlation spectroscopy between the longitudinal and transverse fields at NV_G_ as derived from the results in (b). Red and blue dots, respectively, denote instances where NV_G_ is negative or neutral; ellipses are guides to the eye. (d) Starting from a configuration where both NV_F_ and NV_G_ are negatively charged (blue trace), we probe the cluster response after resonant ionization of NV_G_ (red trace). (e) Same as in (d) but for NV_F_ ionization. The reference frequency in all spectra is 470.470 THz.

Optical excitation is known to cycle the NV charge state between neutral and negative^19^—respectively, NV^–^ and NV^0^—implying that NVs alternatively act as local probes or as point sources of electric field, hence inducing observable spectral shifts in their neighbors. Figures 3a and b illustrate the idea in a new subdiffraction cluster comprising four NVs (D through G). The starting green laser pulse randomly initializes the charge state of each NV, thus making them observable upon resonant excitation. Specifically, the yellow arrows on the right-hand side of Figure 3b highlight instances where the NV_G_^–^ resonances are missing, indicative of green-induced ionization (occurring with ∼20% probability^19,20^). We observe in each case a change in the relative peak amplitudes of NV_F_^–^ as well as a blueshift of its resonance frequencies (orange arrows); indeed, Figure 3c shows seamless correspondence between these shifts and the charge state of NV_G_.

We leverage narrow-band excitation to gain control over the spectral response upon selective NV^–^ ionization. Specifically, we first use weak red light to postselect an instance in which both NV_F_ and NV_G_ are negative (blue trace in Figure 3d), and subsequently tune the laser frequency and amplitude to ionize only NV_G_.^20^ Following a second spectral sweep, the concomitant spectral changes we induce in NV_F_ become apparent (red trace). The converse experiment—where we probe NV_G_ upon selectively ionizing NV_F_, Figure 3e—yields an analogous phenomenology, although the frequency shift is different. This asymmetry stems from the distinct orientations of the transverse background bias fields acting on each NV.

**Figure 4:**
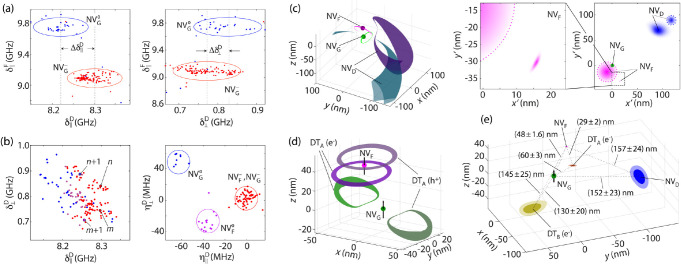
**3D co-localization via multi-NV cross-correlated noise spectroscopy.** (a) Correlations across NV_D_ and NV_F_ as derived from Figure 3b; a charge state change of NV_G_ (red or blue dots) leads to a shift in the mean electric field projections at NV_D_; ellipses are guides to the eye. (b) (Right) Transverse and longitudinal components of the electric field as seen by NV_D_; blue (magenta) dots indicate instances where NV_G_ (NV_F_) is neutral, while red indicates no ionization. Integers *n*, *m* highlight examples corresponding to time consecutive spectra. (Left) Same as before but calculated as δ-field differences η = δ_*n*+1_ – *δ*_*n*_ between two successive measurements; the suppression of slower temporal fluctuations leads to higher spectral resolution. (c) Co-localization of NVs in the cluster of Figure 3. The magenta (green) loop represents the probability distributions for NV_F_ (NV_G_) as seen by NV_G_ (NV_F_). Similarly, the purple (cyan) areas represent probability distributions for NV_D_ using NV_G_ (NV_F_) as the probe; confidence intervals are 70% for NV_D_ and 95% for the rest. (d) Co-localization of dark trap DT_A_, proximal to NV_F_ and NV_G_; upper and lower loops show the 90% probability distributions for the position of the trap as seen by NV_G_ and NV_F_, respectively. (e) (Main) Three-dimensional spatial locations of NV_D_, NV_F_, NV_G_, and proximal traps DT_A_, DT_B_; confidence intervals from dark to light are 50% and 90%. (Upper inserts) Spatial locations of NV_D_, NV_F_, and NV_G_ projected on the *x*′*y*′ optical plane as extracted from the 3D plot. The dashed circles show the NV location measured from super-resolution (95% CI); all locations are relative to NV_G_.

The longer range of Coulombic couplings hints at an interacting cluster, where altering the charge state of one trap ripples on all others. Exposing the effect of weakly coupled NVs, however, is challenging, as background changes in the environment become dominant. We circumvent this complication in Figure 4a where we cross-correlate the δ-fields acting on NV_D_ and NV_F_. We find that the charge state of NV_G_—here serving as a third classifier, blue and red dots—has an impact not only on δ_∥_^F^ but, importantly, on the mean values of δ_∥_^D^ and δ_⊥_^D^, a response that reveals NV_D_’s coupling to NV_G_ (we find a similar response when we exchange the roles of NV_F_ and NV_G_, allowing us to conclude NV_D_ also couples to NV_F_).

Unlike green illumination, weak red excitation has a reduced effect on the charge states of NVs and most traps, suggesting the use of time correlations between consecutive spectral sweeps as a second alternative to mitigating spectral diffusion. Figure 4b shows the field histogram of NV_D_ as well as the difference between the fields measured via successive spectral sweeps in the absence of NV charge initialization by green light. This time-correlation measurement effectively suppresses slow background spectral diffusion and sharpens clusters created by light-induced changes in the charge states of NV_F_ and NV_G_ (SI, Sections 7 and 8). The flipside is a longer experimental time and a partial loss of information as the population statistics of a trap cannot be determined.

We now combine the information gathered thus far to map the NV cluster in three dimensions (Figure 4c). Briefly, we implement a search algorithm that tests all positions of the NVs with respect to each other using measurements from the field histograms of NV_F_ and NV_G_ to calculate the relevant probability distributions (SI, Section 9). Figure 4c illustrates a test performed for the most likely positions (spheres), showing a good intersection between independent measurements of NV_D_ (cyan and purple loops). We estimate the distances of NV_F_ and NV_D_ relative to NV_G_ respectively as (48 ± 1.6) and (150 ± 23) nm, with the greater uncertainty arising from the weaker couplings.

Recurrent optical spectroscopy over an extended data set shows simultaneous shifts of NV_F_ (1.8 GHz) and NV_G_ (600 MHz) approximately 2% of the time, a manifestation of intermittent carrier capture by a trap proximal to both NVs. Here too, we leverage the set of solutions extracted from either NV to co-locate the trap position 29 ± 2 nm from NV_F_ and 60 ± 3 nm from NV_G_ (Figure 4d). The probability distributions do not fully intersect, likely a consequence of the underestimated error sources during data analysis. Changing the nature of the trapped carrier from an electron to a hole leads to disjoint solution sets and can be ruled out (fainter loops in Figure 4d). Co-localization of a trap, therefore, lifts ambiguities in the captured charge sign (and amplitude) inherent to single NV probe sensing, in the process providing clues on the physical nature of the trap: Potential candidates include a substitutional nitrogen impurity (transitioning from N^+^ to N^0^) or a lattice vacancy (changing from V^0^ to V^–^).

Figure 4e integrates the findings above into a combined 3D plot that includes all three interacting NVs in the cluster and dark trap DT_A_, as well as a second trap, DT_B_, observed to capture an electron ∼19% of the time (seemingly sensitive to the charge state of NV_F_, SI, Section 10); this occupancy is higher than that of DT_A_ (2%) and hints at a different physical nature. To compare the cluster geometry against that derived from optical imaging, we leverage the known NV orientations while adjusting the transverse strain axis to determine the transformation matrix connecting the *xyz* frame in the figure to the *x*′*y*′*z*′ reference frame in the lab. Projecting the calculated positions for NV_D_, NV_F_, and NV_G_ onto the *x*′*y*′-plane—perpendicular to the incoming laser beam—yields an image consistent with that attained via super-resolution microscopy (Figure 4e, upper insert).

## Discussion and Outlook

While NV centers are key to the present findings, most ideas can be extended to other color centers in diamond or alternative material hosts: Examples include the silicon vacancy^21^ and carbon–silicon divacancy^22^ in SiC, group-IV vacancy color centers in diamond,^23−27^ emitters in 2D materials,^28^ and rare earth ions in garnets. One can envision methodological extensions in the form of protocols adapted to investigating the trap response under optical excitation not affecting the NV charge state or, alternatively, tailored to probing the diffusion of electrons injected from proximal NVs.^29,30^ Establishing correlations in the fluctuations of optical resonances could prove valuable on its own, e.g., to improve spectral resolution or to characterize new types of emitters.^11,31^ On the other hand, the ability to locate proximal traps hinges on Hamiltonians quantitatively capturing the impact of electric fields on optical resonances, presently available only for select color centers.^12,32,33^

Our approach facilitates the characterization of the microscopic mechanisms underlying spectral diffusion, and hence promises opportunities to developing novel schemes for electric field sensing,^34^ or for tasks in quantum sensing and quantum information processing.^35−37^ Along related lines, the comparatively long range of Coulombic couplings could be exploited to mediate interactions between spin qubits otherwise too far from each other to couple magnetically.^38,39^ Besides applications in quantum science and technology, future material science studies will be required to shed light on the formation of NV clusters—observed in our crystals with unanticipated frequency—as an intriguing alternative to implanting nitrogen-rich moieties.^40^

## Data Availability

The data that support the findings of this study as well as the MATLAB source codes for data analysis and 3D reconstruction are available from the corresponding author upon reasonable request.

## Supporting Information Available

Details on the experimental setup, measurement protocol and conditions, assignments of optical resonances, automated analysis of consecutive spectra as well as additional information on the formalism for 3D localization of the cluster NVs and proximal charge traps (PDF)
