# Supplementary material for: Correlated Spectroscopy of Electric Noise with Color Center Clusters
Source: Nano Lett. 2024 May 20;24(22):6474–9. doi: 10.1021/acs.nanolett.4c00222 (PMC11157654; doi:10.1021/acs.nanolett.4c00222)
Supplement: Supplementary file 1 — nl4c00222_si_001.pdf [file nl4c00222_si_001.pdf]

Supplementary Information for

Correlated spectroscopy of electric noise with color center clusters

Tom Delord[1], Richard Monge[1], and Carlos A. Meriles[1,2,*]

[1]Department. of Physics, CUNY-City College of New York, New York, NY 10031, USA.
[2]CUNY-The Graduate Center, New York, NY 10016, USA.

[*]E-mail: cmeriles@ccny.cuny.edu

1. Experimental

We make use of a custom-made confocal microscope integrating a narrow-linewidth (500 kHz), 637-nm laser tunable across a 4 nm range (Toptica DL PRO), and two off-resonant, continuous-wave (cw) 532-nm lasers. We combine all light sources into a single-mode fiber and use a 605-nm dichroic mirror and a 70/30 beam splitter to selectively collect the sample fluorescence; we control the sample temperature via a closed-cycle cryo-workstation from Montana Instruments[9,1]. Acousto-optic modulators (AOM) in a double-pass configuration produce 10-ns-risetime laser pulses for the 637-nm laser as well as one of the 532-nm lasers (Coherent Sapphire 532-100 CW SF).

To avoid green bleed-through during a low-power red scan, we replace the AOM-controlled 532-nm laser by a 532-nm laser diode (Thorlabs DJ532-40), which we then use for NV charge initialization (with typical power of 200 μW). We carry out NV excitation and photoluminescence (PL) collection via a 0.75-NA Zeiss objective sitting on a room temperature (RT) platform within the cryo-workstation main chamber; all experiments are performed at 9 K. Two signal generators (Rhode & Schwarz and Stanford Research Systems) serve as the source of microwave (MW), which we deliver through a 25-μm wire overlaid on the sample; we use switches from Minicircuits to produce MW pulses, and implement time-resolved protocols with the help of a pulse generator (SpinCore's Pulse-Blaster).

Throughout our experiments, we study two electronic-grade, [100] diamond crystals purchased from Element 6[2,3] and Delaware Diamond Knives[3], both with approximately 5 ppb of nitrogen content. We probe naturally occurring NV clusters ~5 μm deep in the bulk.

2. Determining the NV spatial orientation

To determine the orientation of all NV's with respect to the laboratory frame, we make use of a permanent magnet outside the cryo-workstation to create a weak magnetic field B at the sample site. The field orientation was determined precisely from the optically-detected magnetic resonance (ODMR) spectra of three crystallographically inequivalent NVs[4]. For the NVs of a cluster, we carry out selective ODMR measurements via resonant red readout[3]. Combining the observed frequencies with the measured field magnitude and orientation, we extract the physical lab frame orientation of each NV resorting to known protocols[4,5]. Relevant to the results in Fig. 4 of the main text, we find that NV_E, NV_F, and NV_G share the same orientation, while NV_D points along a different crystallographic axis.

3. PLE measurements and selective NV^- ionization

We implement two alternative methods: The first one — referred to as "repump PLE"[6] — alternates a green pulse (532 nm, 1 mW, 1 μs, Coherent Sapphire) and a red pulse (637 nm, 500 nW – 4 μW, 500 ns), respectively used to initialize the NV charge state and subsequently probe the NV fluorescence at a given wavelength (Supplementary Fig. 1a). For each laser wavelength in the scan, we typically repeat this protocol 10^5 times; note that green illumination not only impacts the NV charge but also the charge state of the environment, implying the resulting PLE spectrum amounts to an average over all possible configurations.

Supplementary Fig. 1b lays out the second approach, here referred to as "repump-free PLE". This protocol starts with a single green pulse (532 nm, 200 μW, 1–10 s, Thorlabs DJ532-40), which we use to charge-initialize the NV cluster and spatially track the photoluminescence (thus accounting for long term drifts). We then apply a weak readout pulse (637 nm, 3–10 nW) simultaneously with continuous MW excitation at 2.877 GHz for 1.2–2.4 seconds per wavelength step, absent of any applied magnetic field. The entire protocol takes 10-20 minutes for a ~400 point spectrum.

The experiments in Fig. 3 of the main text rely on selective ionization of a target NV^- in the cluster, which we implement via an adapted version of the repump-free PLE protocol (Supplementary Fig. 2a). Specifically, we first determine the NV charge state via a non-destructive PLE scan using only 3 nW of 637-nm excitation (Supplementary Fig. 2b). We subsequently stabilize the

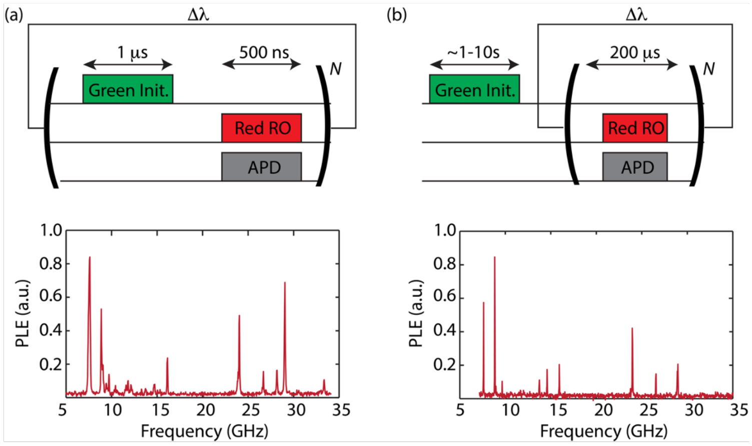

Supplementary Figure 1: PLE acquisition protocols. (a) (Top) Schematics of "repump" PLE. (Bottom) Example NV cluster spectrum upon application of the above protocol. (b) Same as in (a) but for a "repump-free" protocol. By comparison, the absence of charge initialization via a green reset at each wavelength leads to considerably narrower lines, which, nonetheless become subject to spectral diffusion upon repeated observations. In (a) and (b), $\Delta\lambda$ represents the wavelength step during a laser scan, and N is the number of repeats at a given wavelength. The reference frequency in the spectra is 470.470 THz.

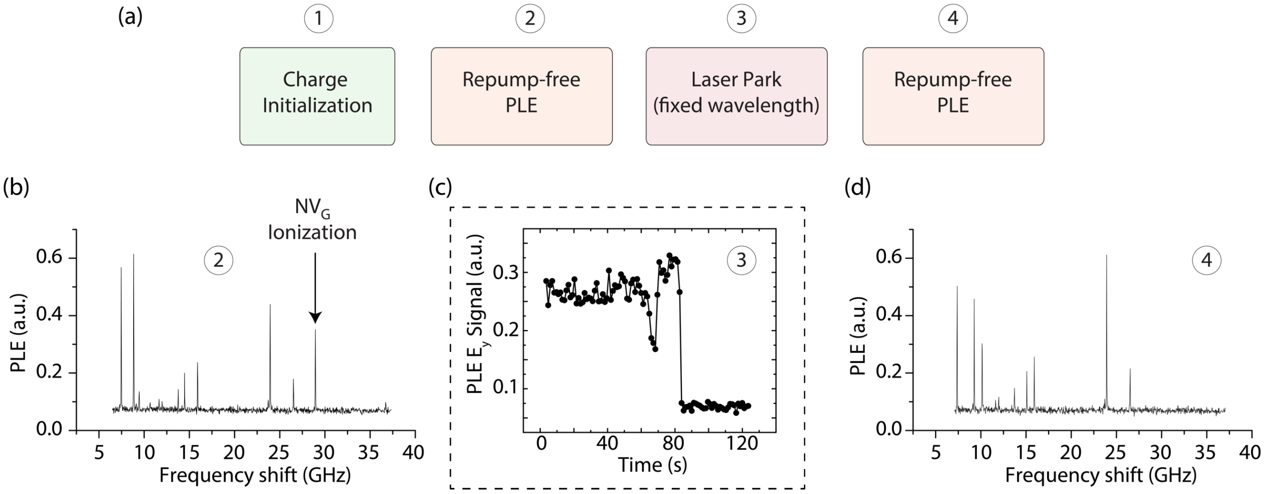

Supplementary Figure 2: NV-selective ionization. (a) Schematics of the protocol. (b) PLE spectrum from the NV cluster in Fig. 3 of the main text prior to selective ionization. The arrow points to the chosen NV$^-$ optical resonance, in this case the E_y transition of NV$_G$. (c) Amplitude of E_y resonance as a function of time; NV$^-$ ionization leads to a sharp drop. (d) Repump-free spectrum of the same NV cluster upon selective ionization of NV$_G$.

red laser at a wavelength resonant with one of the optical transitions of the target NV$^-$, to then monitor the resulting fluorescence under continuous illumination; a sudden signal drop flags ionization (Supplementary Fig. 2c). Lastly, we confirm the procedure acted selectively on the target NV$^-$ via a second non-destructive PLE scan (Supplementary Fig. 2d). Note that red excitation selectively acts on NV$^-$ as the zero-phonon lines of NV^0 lie beyond the tunable laser range[7].

4. PLE assignments and sub-diffraction NV imaging

To establish a relation between the NV eigen-energies and the local strain/electric field δ, we use the expression for the NV excited state Hamiltonian, H_{es}, derived by Rogers et al.[8] Upon numerically diagonalizing this Hamiltonian, we find the δ-dependent eigenvalues presented in the upper plot of Fig. 1a in the main text. To assign each peak in a PLE spectrum, we adapt a repump PLE protocol so as to include MW inversion pulses resonant with the crystal field splitting of the ground state triplet; reversal of the NV$^-$ spin population from $m_S = 0$ — the spin projection after green excitation — to $m_S = \pm 1$ allows us to identify the E_x and E_y resonances via a drop in their PLE amplitudes; with $\delta_\perp = \left(\delta_x^2 + \delta_y^2\right)^{1/2}$ determined from the frequency splitting between these two resonances, all other assignments follow immediately from the set of eigenvalues derived for H_{es}. Note that the expression above assumes $\delta_\parallel = 0$; a non-null value in a measurement amounts to a net shift of all spectral lines relative to a reference.

When working with clusters, we resort to different strategies to confirm the assignments we have made. For example, we implement selective ionization protocols to identify the subset of resonances in the cluster spectrum corresponding to an individual NV within the set. Further, we check the line assignments of a given NV by monitoring the cluster spectrum upon MW-assisted repump PLE: Note that while the E_x and E_y PLE amplitudes fall off upon depleting the $m_S = 0$ spin projection, all others — i.e., E_1, E_2, A_1, and A_2 — must grow as they are preferentially associated to $m_S = \pm 1$ spin

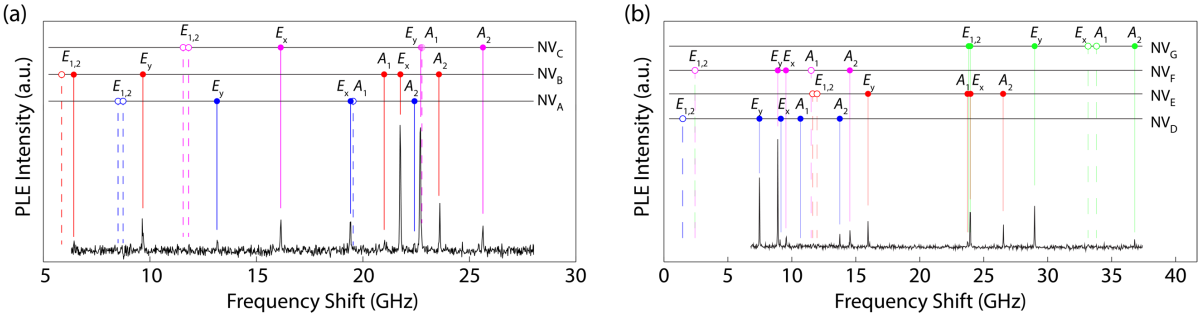

Supplementary Figure 3: Assignment of optical resonances. (a) Repump-free PLE spectrum for the NV cluster in Fig. 1 of the main text under conditions similar to those in that figure. Assignments for each NV follow from combining selective ionization and MW pulse excitation as described in the text. (b) Same as in (a) but for the NV cluster in Fig. 3 of the main text. In (a) and (b), the reference frequency in 470.470 THz.

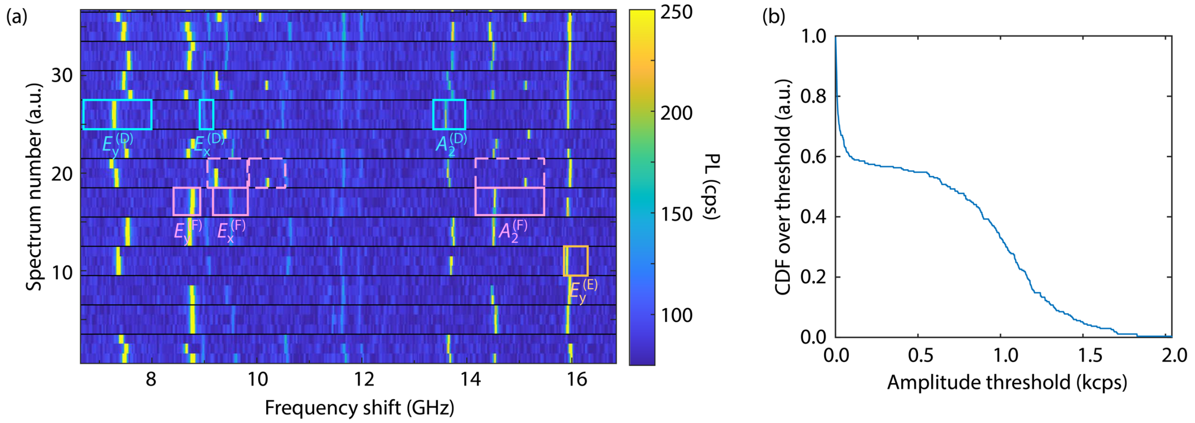

Supplementary Figure 4: Automated analysis of a spectral series. (a) Spectral series for the cluster in Fig 3 of the main text (low frequency end only) illustrating some of the "bands" we use in our code during automatic searches of a given resonance. Colored boxes of indicate different NVs in the cluster. Dotted rectangles indicate secondary spectral bands in NV_F chosen so as to take into account the shift introduced by sporadic ionization of NV_G. Black horizontal lines every 3 spectra denote resets of the NV charge state with green light. The resonant laser power is 3 nW and the PL acquisition time per frequency step is 2 s; the number of points in each spectrum is 700 amounting to an acquisition time of 25 minutes per scan. Frequency shifts are referenced to 470.470 THz. (b) Cumulative distribution function (CDF) upon a Gaussian fit of $E_x^{(F)}$ in the first spectral band (solid pink rectangle). For an amplitude threshold of 0.075 kcps (set by default in our analysis code), we find a PLE line in 61% of the spectra.

states.

We determine the spatial positions of all NVs in a sub-diffraction cluster by implementing confocal imaging under resonant excitation[9]. To this end, we first tune the laser frequency to one of the optical transitions pertaining to the NV of interest (assumed known after proper assignment), and then implement the repump PLE protocol as we galvo-scan the laser across the field of view. We finally determine the NV position with sub-diffraction resolution from a two-dimensional point-spread Gaussian fit of the resulting image.

5. Automated analysis of multiple consecutive spectra

We process all optical spectra in a series via a custom-designed algorithm, the purpose of which is to determine throughout a given frequency scan the spectral positions of at least two optical resonances (including E_x and E_y) for each NV in a cluster. A prerequisite in implementing this protocol is the manual assignment of all resonances (see Section on PLE assignment above), which we then leverage to properly configure our code for automated identification across a spectral series. In particular, we rely on the early assignments and thorough visual inspection of the spectral series to preset "search bands" for each resonance, i.e., spectral windows where we anticipate finding a PLE line (Fig. S3a). Note that for NVs known to experience large discrete jumps (e.g., NV_F and NV_G in the cluster of Fig. 3 in the main text), more than one band might be assigned to a given transition (see solid and dashed pink rectangles in Supplementary Fig. 4a). Whenever two resonances (often belonging to two different NVs) overlap, we make use of other, more isolated optical transitions of the same NV to narrow down the likely frequencies (e.g., we use the strong correlation between the A_2 and E_x resonances to identify the approximate frequency of transition E_x, even when proximal to a resonance from another NV). We extract the central frequency, width, and amplitude of each PLE line from a Gaussian fit, which we activate only when the observed PLE amplitude exceeds a predefined threshold (Supplementary Fig. 4b).

For a given set of NV resonances, we determine $\delta_\perp$ from the set of eigenenergies derived from diagonalizing H_{es}. In principle, any one pair of two lines suffices, but some pairs are more sensitive (e.g., E_x, E_y) than others (e.g., A_1, A_2). When relying on more than two resonances, we ensure optimal accuracy by first calculating the electric field from every combination of PLE lines, and subsequently determining an average weighted by the derivative of the extracted electric field relative to the frequency difference between the corresponding resonances. For each NV in a cluster, we use the same number of resonances — 2 to 4 depending on the NV and experiment — to determine the electric fields for all spectra in the series.

While $\delta_\perp$ relates to the *frequency differences* between resonances, we extract $\delta_\parallel$ by comparing the *average frequency* in each NV spectrum to a fixed reference; note this reference can be arbitrary as our measurements are only susceptible to electric field changes (i.e., we cannot determine the absolute number of elementary charges in a trap). Further, charges of opposite signs lead to different spatial probability distributions, hence creating an ambiguity that can only be lifted by a co-analysis of the

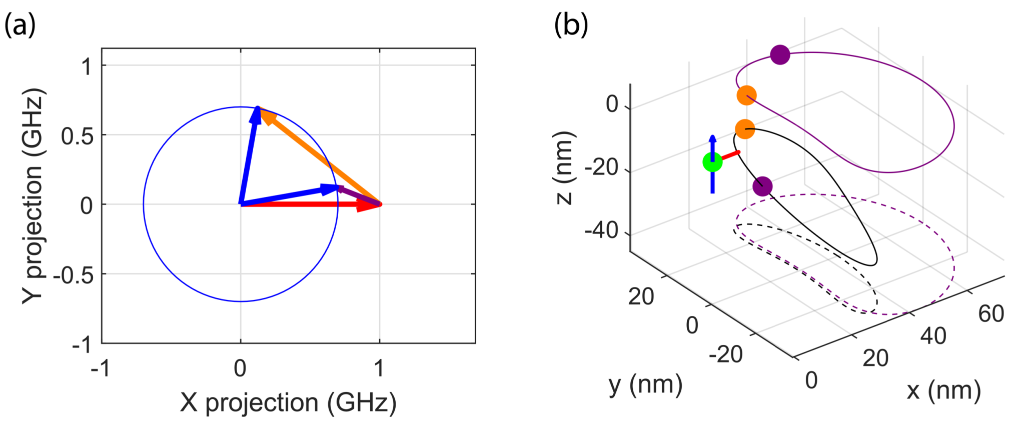

Supplementary Figure 5: Illustrating the impact of an underlying bias field. (a) Effect of the bias field (red arrow) on the measurement of the transverse field produced by an added charge. Since a measurement determines only the magnitude $\delta_\perp$ (blue circle), different fields (e.g., purple and orange arrows) can both lead to the same end observation despite their different magnitude and orientation (blue and light-blue arrows). (b) Effect on the localization of a discrete charge trap. The purple and orange circles are the positions corresponding to the purple and orange arrows in (a), matched by color. The purple (black) line shows all possible positions for a negative charge to generate an end transverse field with the magnitude shown in (a) without (with) an identical change of longitudinal field (300 MHz). In this schematic, the green dot represents the NV at the origin, the red arrow points in the direction of the bias transverse field, and the blue arrow represents the direction of the NV crystal axis.

field fluctuations experienced by a second probe NV. We return to this point below.

When analyzing the impact of a given NV (later referred to as the "source" NV) on another one in the cluster (the "probe" NV), we rely on the presence or absence of a reference optical line in the source NV spectrum to flag its charge state during a PLE sweep. Since spectral scans proceed from lower to higher frequencies, we choose this reference as the highest-energy optical transition in the source NV spectrum so as to mitigate errors arising from mid-scan ionization.

Throughout the manuscript, we express δ-fields (i.e., strain/electric fields) in GHz. To convert between units, we use the proportionality factor $\mu_{\rm E} = 6.3\,\frac{\rm GHz}{\rm MV\,m^{-1}}$, corresponding to a dipole moment difference $\Delta\mu = 1.3$ Debye between the NV$^-$ ground and excited states[10]. Interestingly, this value is not known with accuracy[11,12-14], and could be re-derived by combining the present technique with a super-resolution measurement of two NVs in the same plane.

6. Charge trap mapping via a single NV

Let us consider an individual charge trap near a probe NV and assume we can measure its charge state. By studying the NV field histogram, we can calculate the average magnitude of the electric field transverse and longitudinal components at the NV site depending on the charge state of the trap, which allows us to set boundaries on the trap's spatial location. Since we only determine the transverse field magnitude and not its orientation, the angle θ describing the transverse electric field rotation upon an electric field change remains undetermined, implying the solution takes generically the form of a closed loop in 3D space (Supplementary Figure 5).

More formally, let $\mathcal{E}_\parallel$, $\mathcal{E}_\perp$ (respectively, $\Upsilon_\parallel$, $\Upsilon_\perp$) be the longitudinal and transverse fields with (without) an added charge q. Assuming the NV sits at the origin, we find that for a given rotation θ of the transverse field, the charge trap must be located at a position $R(\theta, \mathcal{E}_\parallel - \Upsilon_\parallel, \mathcal{E}_\perp, \Upsilon_\perp)$ given by

$$\boldsymbol{R} = \left(\frac{q}{4\pi\epsilon_d}\right)^{1/2} \frac{\boldsymbol{\mathcal{E}_c}}{|\boldsymbol{\mathcal{E}_c}|^{3/2}}, \qquad (1)$$

Where ϵ_d is the dielectric constant of diamond and $\boldsymbol{\mathcal{E}_c}$ denotes the field due to q, here expressed as

$$\boldsymbol{\mathcal{E}_c} = \begin{pmatrix} \mathcal{E}_\perp \cos(\theta) - \Upsilon_\perp \\ \mathcal{E}_\perp \sin(\theta) \\ \mathcal{E}_\parallel - \Upsilon_\parallel \end{pmatrix}. \qquad (2)$$

The above formula for the trap position $\boldsymbol{R}$ is only valid for exact measurements, and we must in practice deal with the uncertainty created by other fluctuations of the field or by experimental noise. We now focus on calculating the conditional probability distribution $P(\boldsymbol{r}|\boldsymbol{M})$ of finding a charge at position $\boldsymbol{r}$ given a measurement $\boldsymbol{M} = (\Delta\mathcal{E}_\parallel, \mathcal{E}_\perp, \Upsilon_\perp)$ with variance $\boldsymbol{\sigma}^2_{\mathbf{M}} = \left(\sigma^2_{\Delta\mathcal{E}_\parallel}, \sigma^2_{\mathcal{E}_\perp}, \sigma^2_{\Upsilon_\perp}\right)$, where $\Delta\mathcal{E}_\parallel = \mathcal{E}_\parallel - \Upsilon_\parallel$, and σ^2_ν denotes the variance for variable ν. We follow two alternative routes to calculate $P(\boldsymbol{r}|\boldsymbol{M})$, each featuring complementary computational speed and accuracy. Our first strategy determines the most likely position of the trap following a Bayesian approach. Let us assume a single charge is within a certain volume V of the NV center. A measurement $\boldsymbol{M}$ (with associated variance

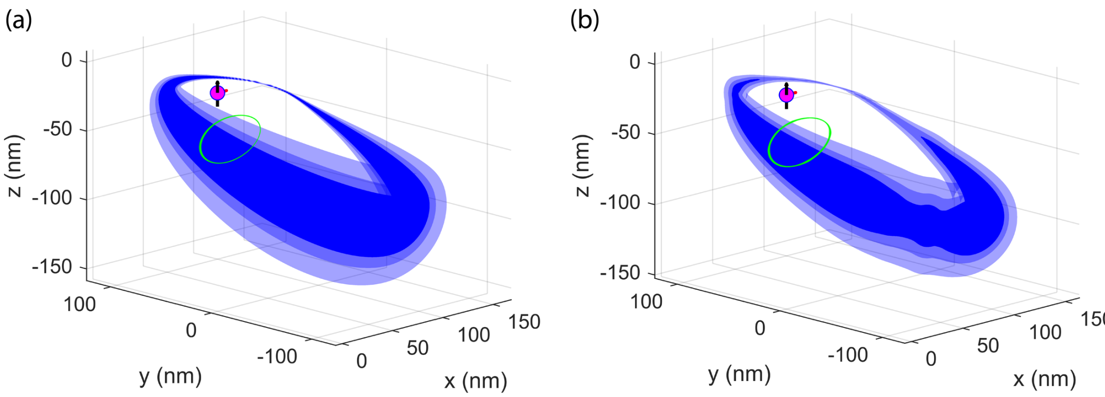

Supplementary Figure 6: Probability distribution for NV_G and NV_D (green and blue loops, respectively) as determined from using NV_F as the probe (magenta circle) using a Bayesian approach; the blue arrow indicates the direction of the crystal field at NV_F. (b) Same as in (a) but using Gaussian error propagation; the result closely reproduces that in (a) but demands a shorter computing time.

$\boldsymbol{\sigma_M^2}$) has been carried out, which informs us on the charge position. Using Bayes theorem, we then write

$$P(\boldsymbol{r}|\boldsymbol{M})P(\boldsymbol{M}) = P(\boldsymbol{r})P(\boldsymbol{M}|\boldsymbol{r}). \qquad (6)$$

Assuming no prior on the charge position, $P(\boldsymbol{r})$ is a constant thus allowing us to cast $P(\boldsymbol{r}|\boldsymbol{M})$ in the form

$$P(\boldsymbol{r}|\boldsymbol{M}) = \frac{P(\boldsymbol{M}|\boldsymbol{r})}{\iiint P(\boldsymbol{M}|\boldsymbol{r})dr^3}, \qquad (7)$$

where we have used the relation $P(\boldsymbol{M}) = \iiint P(\boldsymbol{M}|\boldsymbol{r}')P(\boldsymbol{r}')dr'^3$ and the integral extends over the diffraction volume. $P(\boldsymbol{M}|\boldsymbol{r})$ can be calculated using the field histogram created by a charge at position $\boldsymbol{r}$ as well as the measurement variance. As an illustration, Supplementary Fig. 6a shows the probability distribution for the positions of NV_G and NV_D as seen by NV_F.

The Bayesian approach above is accurate but too resource intensive for cases where many iterations are necessary (the case in Section 7, below). We can gain computational speed via a simpler strategy that relies on summing many Gaussian distributions obtained by the impact of measurement errors at a given point. We can gain computational speed via a simpler strategy that relies on a Gaussian distribution for all errors. Assuming that the presence of a charge q rotates the transverse field by a known angle θ_k, we calculate the probability $P(\boldsymbol{r}|\boldsymbol{M}, \theta_k)$ from the error propagation formula as the 3D Gaussian distribution

$$G_{\theta_k}(\boldsymbol{r}) = \prod_{\nu=x,y,z} \frac{1}{\sigma_\nu\sqrt{2\pi}} \exp\left(-\frac{(r^\nu - R_k^\nu)^2}{2\sigma_\nu^2}\right), \qquad (3)$$

centered at $\boldsymbol{R_k} = \boldsymbol{R}(\theta_k, \mathcal{E}_\parallel - \Upsilon_\parallel, \mathcal{E}_\perp, \Upsilon_\perp)$ with variances defined as

$$\begin{pmatrix}\sigma_x^2\\ \sigma_y^2\\ \sigma_z^2\end{pmatrix} = \left(\frac{\partial \boldsymbol{R}}{\partial \Delta\mathcal{E}_\parallel}\right)^2 \sigma_{\Delta\mathcal{E}_\parallel}^2 + \left(\frac{\partial \boldsymbol{R}}{\partial \mathcal{E}_\perp}\right)^2 \sigma_{\mathcal{E}_\perp}^2 + \left(\frac{\partial \boldsymbol{R}}{\partial \Upsilon_\perp}\right)^2 \sigma_{\Upsilon_\perp}^2. \quad (4)$$

We then approximate the probability $P(\boldsymbol{r}|\boldsymbol{M})$ in the general case where θ is not known, by performing a sum of the Gaussian distributions for a series of θs, i.e.,

$$P(\boldsymbol{r}|\boldsymbol{M}) = \frac{1}{Z}\sum_k z_k\, P(\boldsymbol{r}|\boldsymbol{M}, \theta_k), \qquad (5)$$

where we introduced the renormalization factor $Z = \iiint \sum_k P(\boldsymbol{r}|\boldsymbol{M}, \theta_k)dr^3$ and $z_k = \sigma_{k,x}\sigma_{k,y}\sigma_{k,z}(2\pi)^{\frac{3}{2}}$. The distribution of θ_ks we use is chosen such that adjacent Gaussian probabilities overlap to the same extent by applying the condition $G_{\theta_k}(\boldsymbol{r} = \boldsymbol{R_{k-1}}) \cong 0.8$. Together with the weight z_k, the latter ensures that the probability distribution $P(R(\theta, \boldsymbol{M})|\boldsymbol{M})$ is equal for any θ.

Supplementary Fig. 6b shows again the probability distributions for NV_G and NV_D when probed by NV_F as derived from Gaussian error propagation. We find the result closely reproduces that obtained via the Bayesian approach while demanding only a fraction of the computational time. We therefore follow one route or the other depending on the complexity of the problem at hand.

7. Drift of the background bias field

The crystal strain and electric field applied to the NV have similar effects[7], here we refer to their added contribution generically as the δ-field. For a repetitive set of experiments, we distinguish the average bias field an NV sees from temporal fluctuations from one spectrum to the next. Consistent with previous observations over longer distances[9,,15], we find that NVs tens of nm from each other experience a local bias field different from one another, even for defects along the same crystalline axis. Interestingly, the average bias field of NVs is not constant and can slowly drift over timescale of several hours to days, as shown in Supplementary Fig. 7 for the NV cluster in Fig. 3 of the main text. Alternative mechanisms could be responsible for these drifts. As observed in Monge et

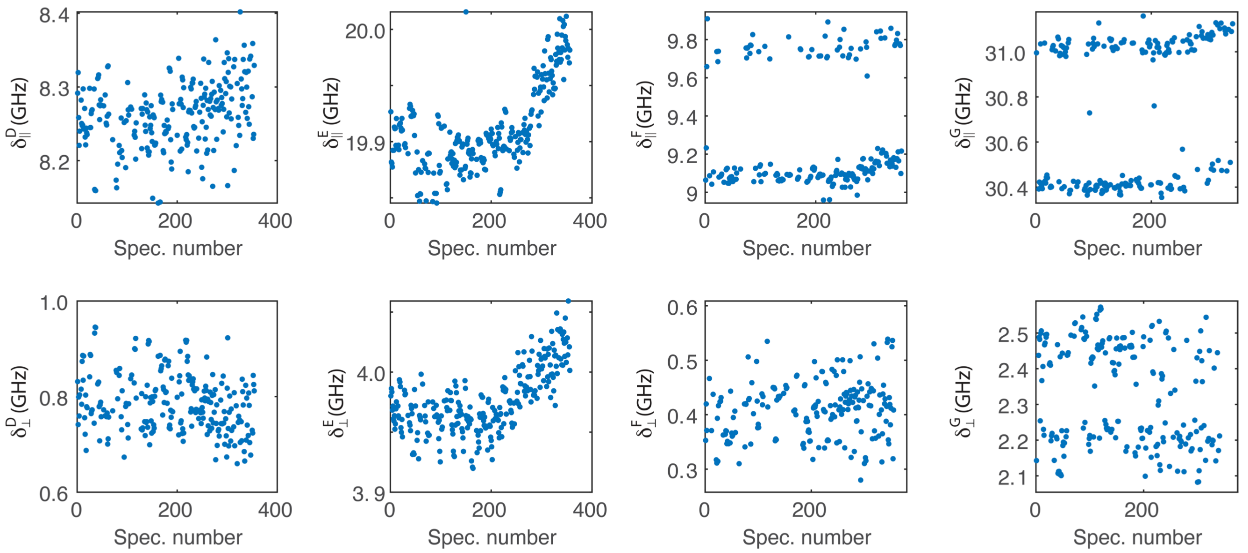

Supplementary Figure 7: Longitudinal and transverse components of the electric fields for the NVs in the cluster of Fig. 3 in the main text during a span of 10 days. A 100 MHz shift of the fields is visible for most NVs. Each spectrum takes 25 min, the average interval between spectra is 40 mins.

al.[9] and consistent with works on bound membranes[16], the diamond attachment method impacts the overall strain in the crystal, implying that minute, slow changes can create drifts. Changes could also be caused by a progressive rearrangement of a metastable electric environment (in the bulk, or on the surface). Indeed, the 100 MHz shift observed here could be induced by a change of one elementary charge at a 150-nm distance, 40 charges at 1 µm, or 1000 charges at 5 µm; such rearrangements have been observed[14]. All field histograms in the manuscript were obtained from spectral series showing no visible drifts. For Fig. 4 of the main text, two datasets obtained months apart and featuring spectral shift differing by hundreds of MHz were used separately to map out all three NVs (and the adjacent dark traps). Importantly, we found the resulting NV localization to be robust from one dataset to another provided we account for a rotation of the transverse field. While these drifts complicate the analysis of field histograms, one could likely apply a correction, or exploit the changing orientation of the transverse field to enhance sensitivity. The same multi-sensor spatial analysis can also be extended to the slower drift to determine their origin.

8. Time-correlation of the electric field

At low enough excitation power, we can perform several repump-free PLE sweeps in a row without green re-initialization of the charge environment. This opens the prospect of studying the evolution of the electric environment over time. Here, we recorded a series of 270 spectra under 3 nW of red excitation with one repump every three spectra. Note that under these parameters, the probabilistic ionization of the NVs we read out remains important. This could be overcome by using a combination of higher NA, lower power, lower detector dark count and higher acquisition time. For each three-PLE-sweep set, we track the electric field shift between consecutive spectra as well as the evolution of the charge state of all NVs. Fig. 4b of the main text shows the field shift histograms we measure. We observe this time-evolution of the electric environment under resonant excitation is slow such that the field shift histograms are dominated by NV ionization events. By measuring the field shifts instead of the absolute value of the electric field, we negate background contributions that do not vary, which improves the minimum shift we can measure and increases the sensitivity and range of our sensor.

This scheme can be generalized to charge traps responding to green illumination by lowering the intensity or duration of the green laser pulse such that it alters the charge of no more than one dark trap[17]. In the present experiment, the green pulses are large enough to fully scramble the environment, such that little to no correlation can be seen between two spectra each preceded by a green reset.

9. Localization using a tri-partite interaction

In general, one can use the multipartite interaction in a cluster of more than two NVs to infer their relative positions and local bias fields. In the present case, we developed an algorithm adapted to 3 NVs, two of whom have the same crystalline axis (NV_F and NV_G in the cluster of Figs. 3 and 4 of the main text). As a brief overview, we first assume NV_G is at the origin, and set its crystalline (bias δ-field) axis along z (x). We then proceed to calculate the probability $P(r)$ for the relative positions of NV_D and NV_F, respectively $\mathbf{r}_D$ and $\mathbf{r}_F$, as well as the relative angle ϕ_F between the transverse bias fields of NV_F and NV_G

(i.e., the angle between the average of $\boldsymbol{\delta}_{\perp}^{\mathrm{NV_F}}$ and $\boldsymbol{\delta}_{\perp}^{\mathrm{NV_G}}$). Using the shortened notation $r = (\mathbf{r}_\mathrm{D}, \mathbf{r}_\mathrm{F}, \phi_F)$, we subsequently assess the probability $P(r)$ by sequentially combining measurements obtained from the field histograms of $\mathrm{NV_F}$ and $\mathrm{NV_G}$. Throughout our analysis, we use each successive measurement to eliminate unlikely values of r and accelerate the search. The resulting probability density is then projected on the $\mathbf{r}_\mathrm{D}$ and $\mathbf{r}_\mathrm{F}$ subspaces, yielding probability densities for those two NVs and an average value for $\phi_F = 80 \pm 20°$.

In the following, we refer to the NVs generating and sensing the electric field as the "source" and "probe" NVs, respectively. The steps below describe the search algorithm in further depth.

1 – For all combinations of source and probe NVs, respectively NV_α and NV_β, $\alpha, \beta = \mathrm{D, E, F, G}$, we calculate the probability distribution for NV_α relative to NV_β as the sum of Gaussian distributions $\sum_k G_k^{\alpha,\beta}$, where $G_k^{\alpha,\beta}$ is centered at $\boldsymbol{R}_{\boldsymbol{k}}^{\boldsymbol{\alpha,\beta}} = \boldsymbol{R}\left(\theta_k^{\alpha,\beta}, \Delta\mathcal{E}_\parallel^{\alpha,\beta}, \mathcal{E}_\perp^{\alpha,\beta}, \Upsilon_\perp^{\beta}\right)$ with standard deviation $\sigma_k^{\alpha,\beta}$, and the sum extends over a discrete set of k positions along the solution loop in 3D space. We also calculate the electric field $\boldsymbol{\mathcal{E}_c}\left(\alpha, \beta, \theta_k^{\alpha,\beta}\right)$ that NV_α at position $\boldsymbol{R}_{\boldsymbol{k}}^{\boldsymbol{\alpha,\beta}}$ would exert on NV_β.

2 – For each probe NV_β, and for every pair of source NVs, NV_α and NV_γ, $\alpha, \beta, \gamma = \mathrm{D, E, F, G}$, we iterate through all Gaussian distributions of $\boldsymbol{G}_{\boldsymbol{k}}^{\alpha,\beta}$ and $\boldsymbol{G}_{\boldsymbol{k'}}^{\gamma,\beta}$ describing the positions of NV_α and NV_γ to calculate the transverse electric fields created by only one NV, $\boldsymbol{\mathcal{E}}_{c,\perp}\left(\alpha, \beta, \theta_k^{\alpha,\beta}\right)$ and $\boldsymbol{\mathcal{E}}_{c,\perp}\left(\gamma, \beta, \theta_{k'}^{\gamma,\beta}\right)$, and by the sum of both, $\boldsymbol{\mathcal{E}}_{\boldsymbol{\Sigma},\perp}$. Since the transverse field is a vector, we have:

$$\left|\boldsymbol{\mathcal{E}}_{\boldsymbol{\Sigma},\perp}\left(\alpha, \beta, \gamma, \theta_k^{\alpha,\beta}, \theta_{k'}^{\gamma,\beta}\right)\right| = \left|\boldsymbol{\Upsilon}_\perp + \boldsymbol{\mathcal{E}}_{\boldsymbol{c},\perp}\left(\alpha, \beta, \theta_k^{\alpha,\beta}\right) + \boldsymbol{\mathcal{E}}_{c,\perp}\left(\gamma, \beta, \theta_{k'}^{\gamma,\beta}\right)\right|. \quad (8)$$

We compare the calculated values for $\boldsymbol{\mathcal{E}}_{\boldsymbol{\Sigma},\perp}$ with that obtained experimentally from the field histogram $\boldsymbol{\mathcal{E}}_{\Sigma,\perp}^{\exp}$, recording the mismatch normalized by the experimental standard deviation, namely, $Z_{\Sigma,\perp}\left(\alpha, \beta, \gamma, \theta_k^{\alpha,\beta}, \theta_{k'}^{\gamma,\beta}\right) = |\mathcal{E}_{\Sigma,\perp}\left(\alpha, \beta, \gamma, \theta_k^{\alpha,\beta}, \theta_{k'}^{\gamma,\beta}\right) - \mathcal{E}_{\Sigma,\perp}^{\exp}|/\sigma_\Sigma$. We then use $Z_{\Sigma,\perp}$ to discard the unlikely $\theta_k^{\alpha,\beta}, \theta_{k'}^{\gamma,\beta}$ pairs (typically $Z_{\Sigma,\perp} > 2$), and subsequently weigh the probability densities of the remaining ones.

3 – We test the most likely relative position between two NVs of the same orientation, NV_α and NV_β. To do so, we iterate through all Gaussian distributions $\boldsymbol{G}_{\boldsymbol{k}}^{\alpha,\beta}$ and $\boldsymbol{G}_{\boldsymbol{k'}}^{\beta,\alpha}$. Note that crystallographically equivalent NVs do not necessarily experience local (transverse or longitudinal) δ-fields of the same orientation. We therefore also test the two possible longitudinal strain directions and sweep the angle $\phi_m^{\alpha,\beta}$ between the transverse strain of the two NVs. For every degeneracy of parameters $\boldsymbol{P} = \theta_k^{\alpha,\beta}, \theta_{k'}^{\gamma,\beta}, \phi_m^{\alpha,\beta}$ we calculate $\boldsymbol{Z}_{k,k',m}^{\alpha,\beta} = |\boldsymbol{R}_k^{\alpha,\beta} - \boldsymbol{O}_{\phi_m^{\alpha,\beta}}(\boldsymbol{R}_{k'}^{\beta,\alpha})|.\left({\boldsymbol{\sigma}_k^{\alpha,\beta}}^2 + \boldsymbol{O}_{\phi_m^{\alpha,\beta}}({\boldsymbol{\sigma}_{k'}^{\beta,\alpha}}^2)\right)^{-1/2}$ and discard unlikely degeneracies when $\min(\boldsymbol{Z}_{k,k',m}^{\alpha,\beta}) > 2$. Here, $\boldsymbol{O}_{\phi_m^{\alpha,\beta}}$ describes the rotation due to the change in transverse strain axis between the two NVs. All remaining pairs of Gaussian distributions are multiplied, i.e., ${\boldsymbol{G}'}_{k,k',\phi}^{\alpha,\beta} = \frac{1}{F_{k,k',\phi}}.\boldsymbol{G}_k^{\alpha,\beta}(\boldsymbol{r}).\boldsymbol{G}_{k'\phi}^{\beta,\alpha}(-\boldsymbol{r})$, which results in a new normal Gaussian distribution of lower amplitude (with $F_{k,k',\phi}$ the renormalization factor); note this distribution has, in general, a different center and standard deviation, which we denote $\boldsymbol{R}_{k,k',\phi}^{\alpha,\beta}$ and $\boldsymbol{\sigma}_{k,k',\phi}^{\alpha,\beta}$.

4 – For every parameter set θ_k, $\theta_{k'}$, ϕ_m, we then calculate the position distribution of a third NV in two independent ways: directly relative to NV_α, and using the relative positions of NV_α and NV_β, $\boldsymbol{R}_{k,k',\phi}^{\alpha,\beta}$, as well as the known distribution of position for NV_γ relative to NV_β. We proceed similarly to step 3, i.e., we discard cases where the mismatch of the two calculated positions is large compared to the combined variances, and multiply the remaining Gaussian distributions to find a new one for the position of NV_γ relative to NV_β. We determine a new renormalization factor for each distribution.

5 – We sum all Gaussian distributions for the positions of NV_α and NV_γ relative to NV_β, weighing each with their two renormalization factors. We finally renormalize each of the two distributions for NV_α and NV_γ.

10. Co-localization of dark charge traps

As seen in Fig. 2, the NV cluster in Fig. 3 of the main text also contains non-fluorescent (i.e., "dark") charge traps. In an extended dataset partially shown in Supplementary Fig. 8, we observe discrete shifts of the resonances of both $\mathrm{NV_F}$ and $\mathrm{NV_G}$, which we attribute to carrier capture by a dark trap $\mathrm{DT_A}$. These shifts occur 5 times in a series of 225 spectra (2.2±1%). Note that we observed a distinct, stronger shift with similar occurrences that could not be analyzed due to the limited range of our laser sweeps. In addition, the field histogram of $\mathrm{NV_G}$ displays discrete jumps that do not correlate with the charge state of any other NV and that we attribute to carrier capture by a second dark trap, $\mathrm{DT_B}$. On average, these shifts occur in 19% of all spectra (13% and 25% when $\mathrm{NV_F}$ is in its negative and neutral state, respectively). Once a trap is identified, we calculate the probability density for

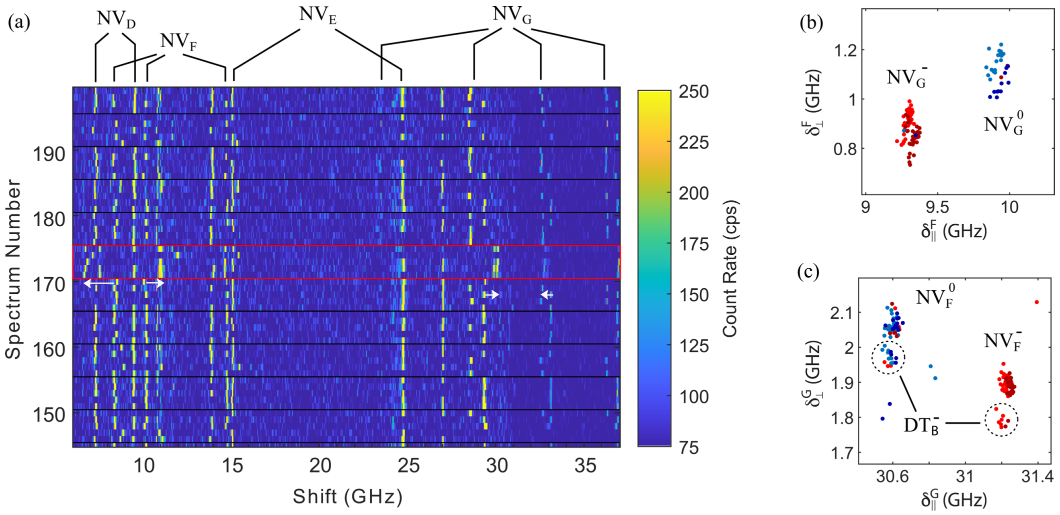

Supplementary Figure 8: (a) Recursive optical spectroscopy of the NV cluster in Fig. 3 of the main text from an extended dataset with a modified strain. We identify discrete shifts of NV$_F$ and NV$_G$ resonances for the five spectra in the red square, as highlighted by the white arrows. (b) Field histograms for NV$_F$. Red and blue dots indicate the charge state of NV$_G$, whereas light and dark shades label the charge state of NV$_D$. (c) Same as in (b) but after exchanging the roles of NV$_G$ and NV$_F$. While the shift due to the DT$_A$ is out of range, we find a discrete shift of the electric field at NV$_G$, which we attribute to a second dark trap, DT$_B$ (dashed circles). The green (red) laser power during charge initialization (frequency sweep) is 1 µW (25 nW); a 10-s, 200-µW green pulse is used for alignment every 5 spectra (black lines in (a)) and the reference frequency is 470.470 THz.

its location relative to NV$_F$ and NV$_G$ assuming a given charge (one electron or one hole). We then use our knowledge of the position and strain of the two NVs to combine that information by simply multiplying and renormalizing the two probability densities (Figs. 4d and 4e in the main text). The relative quality of the intersection of the two loops informs us on the charge sign: By comparing the renormalization factor for a negative and positive charge trap, we find DT$_A$ (DT$_B$) is 10^{300} (10^3) more likely to have one extra electron 2.2% (19%) of the time compared to one hole.

References

[1] R. Monge, T. Delord, N. Proscia, Z. Shotan, H. Jayakumar, J. Henshaw, P. Zangara, A. Lozovoi, D. Pagliero, P.D. Esquinazi, T. An, I. Sodemann, V.M. Menon, C.A. Meriles, "Spin dynamics of a solid-state qubit in proximity to a superconductor", *Nano Lett.* **23**, 422 (2023).

[2] S. Dhomkar, H. Jayakumar, P.R. Zangara, C.A. Meriles, "Charge dynamics in near-surface, variable-density ensembles of nitrogen-vacancy centers in diamond", *Nano Lett.* **18**, 4046 (2018).

[3] R. Monge, T. Delord, G. Thiering, Á. Gali, C.A. Meriles, "Resonant versus non-resonant spin readout of a nitrogen-vacancy center in diamond under cryogenic conditions", *Phys. Rev. Lett.* **131**, 236901 (2023).

[4] T. Weggler, C. Ganslmayer, F. Frank, T. Eilert, F. Jelezko, J. Michaelis, "Determination of the three-dimensional magnetic field vector orientation with nitrogen vacancy centers in diamond", *Nano Lett.* **20**, 2980 (2020).

[5] G. Balasubramanian, I.Y. Chan, R. Kolesov, M. Al-Hmoud, J. Tisler, C. Shin, C. Kim, A. Wojcik, P.R. Hemmer, A. Krueger, T. Hanke, A. Leitenstorfer, R. Bratschitsch, F. Jelezko, J. Wrachtrup, "Nanoscale imaging magnetometry with diamond spins under ambient conditions", *Nature* **455**, 648 (2008).

[6] J.A. Zuber, M. Li, M. Grimau Puigibert, J. Happacher, P. Reiser, B.J. Shields, P. Maletinsky "Shallow silicon vacancy centers with lifetime-limited optical linewidths in diamond nanostructures", arXiv.2307.12753 (2023).

[7] S. Baier, C.E. Bradley, T. Middelburg, V.V. Dobrovitski, T.H. Taminiau, R. Hanson, "Orbital and spin dynamics of single neutrally-charged nitrogen-vacancy centers in diamond", *Phys. Rev. Lett.* **125**, 193601 (2020).

[8] L.J. Rogers, R.L. McMurtrie, M.J. Sellars, N.B. Manson, "Time-averaging within the excited state of the nitrogen-vacancy centre in diamond", *New J. Phys.* **11**, 063007 (2009).

[9] R., Monge, T. Delord, C.A. Meriles, "Reversible optical data storage below the diffraction limit", *Nat. Nanotechnol.* **19**, 202 (2024).

[10] Ph. Tamarat, T. Gaebel, J.R. Rabeau, M. Khan, A.D. Greentree, H. Wilson, L.C.L. Hollenberg, S. Prawer, P. Hemmer, F. Jelezko, J. Wrachtrup, "Stark shift control of single optical centers in diamond", *Phys. Rev. Lett.* **97**, 083002 (2006).

[11] M.W. Doherty, N.B. Manson, P. Delaney, F. Jelezko, J. Wrachtrup, L.C.L. Hollenberg, "The nitrogen-vacancy colour centre in diamond", *Phys. Rep.* **528**, 1 (2013).

[12] Á. Gali, "Ab initio theory of the nitrogen-vacancy center in diamond", *Nanophotonics* **8**, 1907 (2019).

[13] J.R. Maze, Á. Gali, E. Togan, Y. Chu, A. Trifonov, E. Kaxiras, M.D. Lukin, "Properties of nitrogen-vacancy centers in diamond: The group theoretic approach", *New J. Phys.* **13**, 025025 (2011).

[14] L.C. Bassett, F.J. Heremans, C.G. Yale, B.B. Buckley, D.D. Awschalom, “Electrical tuning of single nitrogen-vacancy center optical transitions enhanced by photoinduced fields”, *Phys. Rev. Lett.* **107**, 266403 (2011).

[15] A. Sipahigil, K.D. Jahnke, L.J. Rogers, T. Teraji, J. Isoya, A.S. Zibrov, F. Jelezko, M.D. Lukin, “Indistinguishable photons from separated silicon-vacancy centers in diamond”, *Phys. Rev. Lett.* **113**, 113602 (2014).

[16] X. Guo, M. Xie, A. Addhya, A. Linder, U. Zvi, T.D. Deshmukh, Y. Liu, I.N. Hammock, Z. Li, C.T. DeVault, A. Butcher, A.P. Esser-Kahn, D.D. Awschalom, N. Delegan, P.C. Maurer, F. Joseph Heremans, A.A. High, “Direct-bonded diamond membranes for heterogeneous quantum and electronic technologies”, arXiv:2306.04408 (2023).

[17] W. Ji, Z. Liu, Y. Guo, Z. Hu, J. Zhou, S. Dai, Y. Chen, P. Yu, M. Wang, K. Xia, F. Shi, Y. Wang, J. Du, “Correlated sensing with a solid-state quantum multisensor system for atomic-scale structural analysis”, *Nat. Phot.* **18**, 230 (2024).
